# Combined therapy with methotrexate nanoconjugate and dendritic cells with downregulated IL-10R expression modulates the tumor microenvironment and enhances the systemic anti-tumor immune response in MC38 murine colon carcinoma

**DOI:** 10.3389/fimmu.2023.1155377

**Published:** 2023-03-23

**Authors:** Agnieszka Szczygieł, Katarzyna Węgierek-Ciura, Anna Wróblewska, Jagoda Mierzejewska, Joanna Rossowska, Bożena Szermer-Olearnik, Marta Świtalska, Natalia Anger-Góra, Tomasz M. Goszczyński, Elżbieta Pajtasz-Piasecka

**Affiliations:** Hirszfeld Institute of Immunology and Experimental Therapy, Polish Academy of Sciences, Wrocław, Poland

**Keywords:** immunotherapy, dendritic cells, lentiviral (LV) vector, nanoconjugate, methotrexate, immunomodulation, colon carcinoma, interleukin-10 receptor downregulation

## Abstract

**Background:**

Understanding the negative impact of the tumor microenvironment on the creation of an effective immune response has contributed to the development of new therapeutic anti-cancer strategies. One such solution is combined therapy consisting of chemotherapeutic administration followed by dendritic cell (DC)-based vaccines. The use of cytostatic leads to the elimination of cancer cells, but can also modulate the tumor milieu. Moreover, great efforts are being made to increase the therapeutic outcome of immunotherapy, e.g. by enhancing the ability of DCs to generate an efficient immune response, even in the presence of immunosuppressive cytokines such as IL-10. The study aimed to determine the effectiveness of combined therapy with chemotherapeutic with immunomodulatory potential – HES-MTX nanoconjugate (composed of methotrexate (MTX) and hydroxyethyl starch (HES)) and DCs with downregulated expression of IL-10 receptor stimulated with tumor antigens (DC/shIL-10R/TAg) applied in MC38 murine colon carcinoma model.

**Methods:**

With the use of lentiviral vectors the DCs with decreased expression of IL-10R were obtained and characterized. During *in vivo* studies MC38-tumor bearing mice received MTX or HES-MTX nanoconjugate as a sole treatment or combined with DC-based immunotherapy containing unmodified DCs or DCs transduced with shRNA against IL-10R (or control shRNA sequence). Tumor volume was monitored during the experiment. One week after the last injection of DC-based vaccines, tumor nodules and spleens were dissected for *ex vivo* analysis. The changes in the local and systemic anti-tumor immune response were estimated with the use of flow cytometry and ELISA methods.

**Results and conclusions:**

*In vitro* studies showed that the downregulation of IL-10R expression in DCs enhances their ability to activate the specific anti-tumor immune response. The use of HES-MTX nanoconjugate and DC/shIL-10R/TAg in the therapy of MC38-tumor bearing mice resulted in the greatest tumor growth inhibition. At the local anti-tumor immune response level a decrease in the infiltration of cells with suppressor activity and an increase in the influx of effector cells into MC38 tumor tissue was observed. These changes were crucial to enhance the effective specific immune response at the systemic level, which was revealed in the greatest cytotoxic activity of spleen cells against MC38 cells.

## 1 Introduction

The use of dendritic cells-based vaccines in anti-cancer therapy has been the subject of research for many years. Since the groundbreaking discovery of these cells (1), it has been possible to fully understand their enormous potential in inducing a specific immune response, which allowed for the application of DCs in the treatment of cancer patients (2–4). However, it should be added that the DCs’ capacity to trigger the anti-tumor immune response may be hindered in hostile conditions such as tumor microenvironment (TME) (5). One of the factors preventing the development of a specific immune response is suppressive cytokines such as IL-10, which is largely produced by immune cells infiltrating into the tumor tissue: regulatory T cells (Tregs), tumor-associated macrophages (TAMs) and myeloid-derived suppressor cells (MDSCs) (6–8). It is well known, that IL-10 negatively affects the maturation of DCs, reducing their ability to stimulate a Th1-type immune response (9). Moreover, IL-10-treated DCs show tolerogenic features and induce anergy of antigen-specific CD4^+^ and CD8^+^ T cells (10, 11). The occurrence of this phenomenon in cancer disease may lead to the escape of tumor cells from immune surveillance (12), and the increased concentration of IL-10 in the serum of cancer patients is correlated with an unfavorable prognosis (13–15).

For this reason, great efforts are made to reduce the negative effects of tumor-derived IL-10 on the DC properties. So far, the research carried out in this area assumed the systemic abolition of the activity of this cytokine by using antibodies that neutralize IL-10 (16–18) or block a specific receptor located on the surface of immune cells (19–21). However, the systemic blockade of the activity of IL-10 may lead to the development of autoimmune disorders such as inflammatory bowel disease (22, 23). For this reason, locally acting solutions currently are under investigation. For example, the reduction of IL-10 concentration in TME achieved by intratumoral injections of lentiviral vectors carrying the shRNA sequence directed against IL-10 significantly improved the effectiveness of DC-based vaccines and contributed to the inhibition of the MC38 tumor growth (24). Another approach to enhance the therapeutic effect of DC-based vaccines is the use of DCs with reduced sensitivity to IL-10. By downregulation of specific IL-10 receptor expression on the DCs surface, it is possible to overcome the negative influence of this cytokine on their activity, and it has been investigated *in vitro* (25, 26) and *in vivo* (22).

It should be added that in order to further enhance the effectiveness of DC-based vaccines, the therapeutic schedule should be extended with the administration of cytostatics, which, apart from eliminating cancer cells, can modify the tumor milieu. Preclinical studies that use such a strategy of combined anti-cancer therapy are mainly based on well-known chemotherapeutic agents, such as cyclophosphamide (27, 28) or paclitaxel (29). Nevertheless, an interesting area of research is the use of other commonly used cytostatics, especially when they have been attached to high-molecular carriers. This innovative drug delivery system ensures a more selective drug distribution in the body. An example of such a novel form of chemotherapeutic delivery is the nanoconjugate of methotrexate – one of the oldest antifolate drugs, widely used in anticancer therapy in solid tumors and hematologic malignancies, and hydroxyethyl starch – the amylopectin-based modified polymer applied in medicine as colloidal plasma volume expanders. Through the covalent coupling of MTX and HES, obtained nanoconjugate had a longer plasma half-time compared to the free form of MTX (30), and, importantly – improved drug biodistribution in the body. As a result of the conjugation process, HES-MTX nanoconjugate enters cells *via* folate receptors (FRs), including FRα, which are overexpressed on cancer cells, instead of ubiquitously expressed reduced folate carriers (RFCs), to which MTX in free form has a high affinity (31). Moreover, taking into account the hydrodynamic diameter of the nanoconjugate molecule (15.2 ± 6.2 nm) as well as the abovementioned prolonged plasma half-life, it may be postulated that the HES-MTX nanoconjugate can reach the tumor tissue through an enhanced vascular permeability and retention effect (EPR), which was discussed in our previous publication (32).

Additionally, recently a lot of attention has been focused on the immunomodulatory properties of certain chemotherapeutics. Many reports confirm, that cytostatics, including MTX, can act as modulators of immune cell phenotype but also can provide the favorable stimulation of effector immune cells (33–36). The use of cytostatics with immunomodulatory potential also ensures the temporary depletion of some immune cell populations with suppressor activity, such as MDSCs, TAMs, or Tregs from TME and creates the optimal cytokine environment for DCs to generate the specific anti-tumor immune response (37). The beneficial modulation of immune cells’ landscape in TME is an important factor supporting immunotherapy – especially when chemotherapeutic is applied prior to DC-based vaccines. Thus, considering the abovementioned advantages of using the conjugated form of MTX, it seems reasonable to use HES-MTX nanoconjugate in combination with DC-based vaccines in anticancer therapy.

Therefore, in our previous research, we demonstrated the greater immunomodulatory potential of HES-MTX nanoconjugate composed of hydroxyethyl starch and MTX in contrast to the free form of MTX in MC38 tumor model (32). On the third day after HES-MTX administration, we observed the modulation of tumor milieu through the increasing influx of effector cells and eliminating cells with suppressor activity. These changes were accompanied by the induction of systemic specific anti-tumor immune response. All these alterations led to the creation of appropriate conditions for DCs applied as cellular vaccines to activate the specific immune response against the MC38 tumor. In comparison to the group of mice receiving HES-MTX as sole therapy, the extension of the treatment schedule with multiple peritumoral injections of DC-based vaccines resulted in the enhancement of the local and systemic anti-tumor immune response. However, considering the therapeutic effect of this combined therapy we did not observe the increased tumor growth inhibition, as we initially expected (32). Therefore, we assumed that other tumor suppressor factors are present in the MC38 tumor microenvironment, such as IL-10, which adversely affects the direction and effectiveness of applied DCs.

Considering our previous observations, this study aimed to enhance the therapeutic effect of DC-based vaccines through the downregulation of IL-10 receptor expression on their surface and answer the question whether the modulation of the immune response induced by the administration of MTX-based chemotherapeutic agents influences the effectiveness of those modified DCs.

## 2 Materials and methods

### 2.1 Mice

Female C57BL/6 mice were obtained from the Center of Experimental Medicine of the Medical University of Białystok (Białystok, Poland). Mice were kept in a room with a standard light/dark cycle, with a constant temperature (22 ± 2˚C), air humidity (55 ± 10%) and access to food and water ad libitum. All experiments were performed in accordance with EU Directive 2010/63/EU for animal experiments, and were approved by the Local Ethics Committee for Experiments with the Use of Laboratory Animals, Wrocław, Poland (authorization no. 31/2016 and 068/2020). After the experiments, mice were sacrificed by cervical dislocation.

### 2.2 Cell culture

The *in vivo* growing MC38 murine colon carcinoma from the Tumor Bank of the Radiobiological Institute TNO (Rijswijk, The Netherlands) was adapted to *in vitro* conditions as described by Pajtasz−Piasecka et al. (38). The culture of MC38/0 (named here **MC38**) cells was maintained in RPMI−1640 (Gibco; Thermo Fisher Scientific, Inc.) supplemented with 100 U/ml penicillin, 100 mg/ml streptomycin, 0.5% sodium pyruvate, 0.05 mM 2−mercaptoethanol (named here RPMI) and 5% fetal bovine serum (FBS; all reagents from Sigma−Aldrich; Merck KGaA). Tumor antigen (TAg) was prepared by repeated freezing and thawing of an MC38 cell suspension (5×10^6^ MC38 cells/ml), which was followed by sonication. The Lenti-X 293T cell line (Clontech) was maintained in high-glucose Dulbecco’s Modified Eagle Medium (ATCC) supplemented with 10% FBS. Dendritic cells for *in vitro* and *in vivo* experiments were generated from bone marrow isolated from femurs and tibias of healthy C57BL/6 mice according to the protocol described in the previous publication (39). The cells (named here **DCs**) were cultured in RPMI supplemented with 10% FBS in the presence of recombinant murine (rm)GM−CSF (ImmunoTools, 40 ng/ml) and rmIL−4 (ImmunoTools, 10 ng/ml). After 6 days the loosely attached immature DCs were harvested and placed in T75 cm^2^ flasks at density 15×10^6^ cells/20 ml of RPMI supplemented with 10% FBS, rmGM-CSF (40 ng/ml) and rmIL-4 (5 ng/ml) for subsequent lentiviral transduction. All cell cultures were grown at 37°C, 95% humidity and 5% CO_2_.

### 2.3 Lentiviral vector production

**Figure 1:**
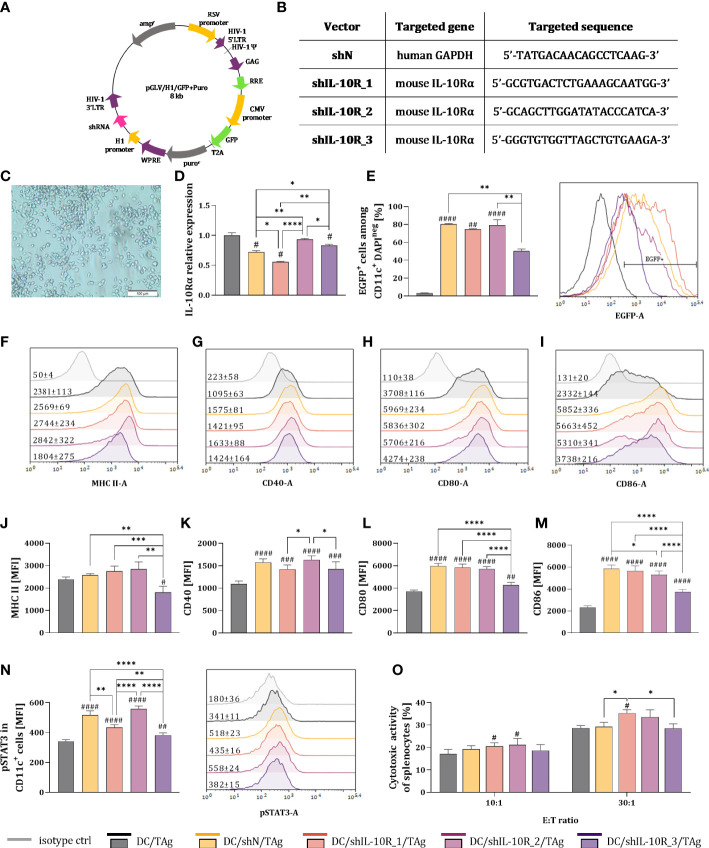
Effectiveness of IL-10 receptor downregulation in bone-marrow derived dendritic cells stimulated with tumor antigens. **(A)** Scheme of 3^rd^ generation lentiviral vector encoding shRNA. **(B)** Targeted nucleotide sequences of selected shRNA against IL-10Rα (shIL-10R) and scrambled control sequence (shN). **(C)** Morphology of DCs stimulated with TAg (Olympus CKX41 light microscope, magnification 12.6x. Scale bar corresponds to 100 µm) **(D)** Relative expression of IL-10Rα mRNA in transduced DCs determined by Real-Time PCR technique. **(E–N)** Bar pots and representative overlay histograms showing the EGFP expression **(E)**, expression of MHC II and costimulatory molecules **(F–L)** and phosphorylated STAT3 after stimulation with rmIL-10 **(N)** gated on CD11c^+^ mature DCs determined by flow cytometry (MFI – mean fluorescence intensity). **(O)** Cytotoxic activity of DCs-activated naïve splenocytes (effector cells) against DiO^+^ MC38 cells (target cells) after 4-hour incubation in 10:1 and 30:1 E:T ratios. Results are expressed as mean + SD calculated for three experiments. On the overlay histograms the numbers represents the average MFI ± SD value for each group. Differences between groups were calculated using **(D, J, N)** the Brown−Forsythe and Welch ANOVA test followed by Dunnett’s T3 multiple comparisons *post−hoc* test; **(K, L, M, O)** one−way ANOVA followed by Tukey’s multiple comparisons *post−hoc* test or **(E)** nonparametric Kruskal−Wallis test followed by Dunn’s multiple comparisons test. The asterisks (*) presented in the graphs indicate statistically significant differences between the given groups; hashtags (#) above a bar indicate a statistically significant difference between the given group and the control group – DC/TAg (*/#p<0.05; **/##p<0.01; ***/###p<0.001; ****/####p<0.0001).

Lentiviral vectors (LVs) were produced using 3^rd^ generation lentiviral system, which consisted of pMDLg/pRRE, pRSV-Rev, pMD2.G plasmids – kindly shared by Didier Trono (Addgene plasmids no. #12251, 12253, 12259), and expression plasmid purchased in EzBiolab (pGLV/H1/GFP + puro^r^). The expression plasmids encoded three different shRNA sequences against the murine alpha unit of IL-10 receptor (shIL-10R) or scrambled control non-targeting sequence of shRNA against human GAPDH (shN) (**Figures 1A, B**). Lentiviral vectors were produced and concentrated according to the procedure described in our previous article (40). Briefly, Lenti-X 293T cells were co−transfected with plasmids and cultured for 48 hours. The supernatant containing lentiviral vectors was collected and concentrated by precipitation using PEG6000 (Sigma−Aldrich; Merck KGaA). The pellet containing lentiviral vectors was suspended in PBS and stored at -80°C. The titer of the lentiviral vectors was determined by the serial dilution method using MC38 cells and flow cytometry analysis.

### 2.4 Preparation of DCs with downregulated IL-10R expression for *in vitro* and *in vivo* experiments

24 hours after placing on the T75 cm^2^ flasks, DCs were transduced with LVs with the assumption, that there were 4 viral infectious particles per 1 dendritic cell in the presence of 8 µg/ml polybrene (Sigma−Aldrich; Merck KGaA) [as described by (41)]. 4 hours after the addition of LVs, DCs were stimulated with tumor antigen lysates (TAg, 10% v/v). 24 hours later DCs were harvested, centrifuged (192 ×g, 7 min, 4°C) and resuspended in the RPMI with 10% FBS, rmGM-CSF (40 ng/ml) and rmIL-4 (5 ng/ml) for subsequent 48 hours. After this time, the transduced DCs stimulated with TAg were collected and applied for *in vitro* characteristics or used as cellular vaccines in *in vivo* experiment described below. Control, non-transduced DC/TAg, cells were maintained analogously to the procedure described above.

### 2.5 Effectiveness of IL-10 receptor downregulation in transduced DCs determined by Real-Time PCR technique

In order to assess the efficiency of IL-10R downregulation in DCs, the determination of *il10ra* mRNA level using real-time PCR technique was performed. Total RNA was isolated using a NucleoSpin RNA kit (Macherey−Nagel) and reverse−transcribed with a First Strand cDNA Synthesis Kit (Thermo Fisher Scientific, Inc.). Real−time PCR was carried out with the use of TaqMan Universal PCR Master Mix and TaqMan Gene Expression Assay primers for IL-10Rα (Mm00434151_m1) in reference to the HPRT gene (Mm00446968_m1; all reagents from Applied Biosystems). The analyses were conducted using the ViiA 7 Real−Time PCR System (Applied Biosystem) with QuantStudio™ Real-Time PCR software.

### 2.6 Phenotype characteristics of transduced DCs with downregulated IL-10R expression by flow cytometry method

In addition to determining the downregulation of expression of the target gene, the efficacy of transduction was assessed by flow cytometry as the percentage of enhanced green fluorescent protein-positive (EGFP^+^) cells. The surface phenotype characteristics of transduced DCs were also evaluated by flow cytometry. For this purpose, cells were stained with anti-MHC II APC-Cy7 (clone M5/114.15.2; BioLegend), anti-CD40 BrilliantViolet 605 (clone 3/23; BD Pharmingen), CD80 PerCP-Cy5.5 (clone 16-10A1; BioLegend), CD86 PE-Cy7 (clone GL-1; BioLegend) and CD11c BrilliantViolet 650 (clone N418, BioLegend) monoclonal antibodies and elimination of the dead cells was carried out by using the DAPI dye.

### 2.7 Determination of intracellular expression of phosphorylated STAT3 protein in DCs with downregulated IL-10R by flow cytometry method

To evaluate the intracellular expression of phosphorylated STAT3 protein, DCs were stimulated with rmIL-10 (1 µg/ml, ImmunoTools) for 15 min followed by cells fixation with the use of Lyse/Fix Buffer (BD Phosflow). Next, fixed cells were permeabilized with Perm Buffer III (BD Biosciences) and stained with anti-STAT3 (pY705) AlexaFluor 647 (clone 4/P-STAT3; BD Phosflow) and CD11c BrilliantViolet 650 (clone N418, BioLegend) antibodies. The flow cytometry analyzes were performed using the LSRFortessa flow cytometer with Diva software (BD Biosciences).

### 2.8 Determination of the ability of DCs with downregulated IL-10R expression to activation of a primary antigen-specific immune response

To evaluate the ability of DC/shIL-10R/TAg to activate the primary antigen For this purpose, the 5-days co-culture of mature DCs (0.18×10^6^ cells) (i.e. stimulated with MC38-derived TAg) and naive spleen cells (splenocytes) (1.8×10^6^ cells) in the presence of recombinant human (rh) IL−2 (200 U/ml, Immunotools) was performed. After this time, cells were harvested and the cytotoxic activity of effector splenocytes against target (MC38) cells stained with DiO lipophilic dye (Molecular Probes) was analyzed according to a previously described procedure (42). Two E:T (effector to target) ratios were investigated: 10:1 and 30:1. The dead target cells were distinguished with propidium iodide (PI) solution and the percentage of DiO^+^PI^+^ MC38 cells was determined using a FACS Calibur with CellQuest 3.3 software (Becton Dickinson).

### 2.9 Therapeutic treatment schedule

Eight-to-ten-week-old female C57BL/6 were subcutaneously (s.c.) inoculated in the right flank with MC38 cells (1.1×10^6^ cells/0.2 ml NaCl 0.9%/mouse). When tumor nodules were palpable, mice were randomly divided on a basis of tumor volume into nine experimental groups with an initial group size of 10-12 mice per group (with the use of open source Randomicer Web Application (https://github.com/mszczygiel/randomicer)). The therapeutic schedule started from intravenously (i.v.) injection in the tail vein of chemotherapeutics – MTX (Ebewe Pharma) or HES-MTX nanoconjugate [HES-MTX preparation was described in (30, 32)] in dose 20 mg/kg body weight. On the 3^rd^, 10^th^, 17^th^ day of therapy, DC-based vaccines (2×10^6^ cells/0.2 ml NaCl 0.9%/mouse/injection) were administered peritumorally (p.t.). In the experiment, three different types of cellular vaccines were used – non-transduced DC/TAg, DCs transduced with control shRNA sequence (DC/shN/TAg) and DCs transduced with shIL-10R_1 shRNA sequence (DC/shIL-10R/TAg). During the experiment, two or three times a week, tumors were measured by using an electronic caliper and tumor volume was calculated according to the formula: *a/2×b^2^*,where *a* represents the largest and *b* represents the smallest tumor diameter (42). The therapeutic efficacy was determined on the basis of the tumor growth inhibition (TGI) value calculated as follows: *TGI*(%)= 100- (*TV_t_/TV_nt_* ×100), where *TV_t_* refers to a median tumor volume in the treated group and *TV_nt_* – median tumor volume in the non-treated (nt) group (39). The health of the mice was constantly monitored (weight loss, bristling hair, lethargy) and when the tumors volume was >2 cm^3^ the mice were sacrificed. In order to estimate the impact of applied therapy on local and systemic anti-tumor immune response, seven days after the last DCs-based vaccine injection, spleen and tumor nodules were dissected from MC38-tumor bearing mice (from 5-8 mice per group), homogenized and stored in liquid nitrogen for further *ex vivo* analyses.

### 2.10 Analysis of myeloid and lymphoid cells in MC38 tumors after therapy by flow cytometry method

Single cell suspensions of tumor tissue were thawed and stained for identification of myeloid and lymphoid cell subpopulations according to the procedure described previously (24). Briefly, tumor suspensions were stained with the LIVE/DEAD Fixable Violet Dead Staining Kit (Thermo Fisher Scientific, Inc.) and then labeled with cocktails of fluorochrome−conjugated monoclonal antibodies: anti−CD3 PE−CF594 (clone 145-2C11), anti−CD19 PE−CF594 (clone 1D3), anti−CD49b PE−CF594 (clone DX5) (all from BD Biosciences), anti−CD45 BrilliantViolet 605 (clone 30-F11), anti−CD11b PerCP−Cy5.5 (clone M1/70), anti−CD11c BrilliantViolet 650 (clone N418), anti−F4/80 AlexaFluor 700 (clone BM8), anti−Ly6C PE (clone HK1.4), anti−Ly6G APC−Cy7 (clone 1A8), anti−MHC II FITC (clone M5/114.15.2), anti−CD80 PE−Cy7 (clone 16-10A1) (all from BioLegend) for myeloid cell identification, and anti−CD45 BrilliantViolet 605 (clone 30-F11), anti−CD3 BrilliantViolet 650 (clone 17A2), anti−CD4 FITC (clone RM4-5), anti−CD8 APC/Fire 750 (clone 53-6.7), anti-CD19 AlexaFluor 700 (clone 6D5), anti−CD25 PE (clone PC61) (all from BioLegend) for lymphocyte identification. Then, the cells were fixed using the Foxp3/Transcription Factor Staining Buffer Set (eBioscience). Cells stained with myeloid or lymphocyte cocktail were additionally incubated with anti−CD206 APC (clone C068C2; BioLegend) or anti−FoxP3 APC (clone FJK-16s; eBioscience) monoclonal antibodies, respectively. The flow cytometry analysis was performed using the LSRFortessa flow cytometer with Diva software (BD Biosciences).

### 2.11 Analysis of the systemic anti-tumor immune response of spleen cells after therapy by flow cytometry method

Spleen single cell suspensions (2×10^6^ cells) were thawed and cocultured with mitomycin C-treated MC38 cells (0.1×10^6^ cells) in the presence of rhIL−2 (200 U/ml). After 5 days of restimulation, supernatants were collected and stored at 4˚C until ELISA was performed. The cytotoxic activity of effector splenic cells against target (MC38) cells was evaluated as described earlier. In order to determine the percentage of CD107a^+^ cells, the degranulation assay was performed. Briefly, restimulated spleen cells were incubated for 2 hours with MC38 cells in the presence of monoclonal anti−CD107a antibody conjugated with APC (clone 1D4B, BioLegend) together with ionomycin (1 µg/ml, Sigma-Aldrich Merck KGaA), phorbol-12-myristate-13-acetate (50 ng/ml, Sigma-Aldrich Merck KGaA) and rhIL-2 (200 U/ml). Afterwards, cells were harvested and stained with anti−CD45 V500 (clone 30-F11), anti−CD4 FITC (clone RM4-5), anti−CD8 PE−Cy7 (clone 53-6.7) and anti−CD49b PE (clone DX5) (all from BioLegend). In order to eliminate the dead cells, DAPI dye was used. The flow cytometry analysis was performed using the LSRFortessa flow cytometer with Diva software (BD Biosciences).

### 2.12 Determination of cytokine production by enzyme-linked immunosorbent assay

Production of cytokines by restimulated spleen cells was evaluated using commercially available ELISA kits (IL−10, IL−4; BD Biosciences and IFN−γ; eBioscience) according to the manufacturer’s instructions.

### 2.13 Statistics

All the data were analyzed using GraphPad Prism 9 software (GraphPad Software, Inc.). The normality of residuals was confirmed by the D’Agostino−Pearson omnibus test. When data were consistent with a Gaussian distribution and had equal SD values, the statistical significance was calculated using the parametric one−way ANOVA followed by Tukey’s multiple comparison *post−hoc* test. When data were consistent with a Gaussian distribution, but SD values were not equal, the Brown−Forsythe and Welch ANOVA test followed by Dunnett’s T3 multiple comparisons *post−hoc* test was performed. Data inconsistent with a Gaussian distribution were analyzed using the nonparametric Kruskal−Wallis test followed by Dunn’s multiple comparison *post−hoc* test. The statistical significance in the kinetics of tumor growth was calculated using the two−way ANOVA followed by Bonferroni’s multiple comparisons *post−hoc* test. The type of statistical analysis used is described in the captions under the figures. All statistically significant differences are presented in the graphs; otherwise, the differences were not significant.

## 3 Results

### 3.1 DCs with downregulated IL-10R expression generated the efficient specific anti-tumor immune response *in vitro*

In the first step of our research, we decided to choose the shRNA sequence that will most effectively reduce the expression of IL-10 receptor in dendritic cells. For this purpose, LVs carrying three different shRNA sequences (designated as shIL-10R_1, shIL-10R_2, shIL-10R_3) directed against the mouse alpha unit of IL-10 receptor were used. Additionally, a negative control sequence (shN) directed against human GAPDH was applied to determine the effect of the viral transduction process on changes in DCs phenotype (**Figures 1A–C**). Considering the *il10ra* mRNA level, the lowest expression of the targeted gene was observed for the shIL-10R_1 sequence. This decrease was statistically significant, not only in relation to control DCs (DC/TAg) or shN sequence but also in comparison to other shRNAs against IL-10R (**Figure 1D**). Based on the percentage of EGFP^+^ cells, it was confirmed that used LVs efficiently transduced CD11c^+^ DCs, however in the case of shIL-10R_3 this percentage was the lowest (**Figure 1E**). Further cytometric analyses revealed that lentiviral transduction caused DCs stimulation, which was reflected especially in the increased expression of costimulatory molecules (**Figures 1F–M**) Among all shIL-10R sequences, the lowest MFI values of tested surface molecules were noted for the shIL-10R_3, which is consistent with the percentage of EGFP^+^ cells in this group. Moreover, to investigate whether the downregulation of IL-10R is related to decreased sensitivity of DCs to IL-10, the assessment of STAT3 phosphorylation has been performed. After stimulation of DCs with rmIL-10, we observed that in DC/shIL-10R_1/TAg and DC/shIL-10R_3/TAg groups the expression of pSTAT3, on the one hand, was higher than the level observed in the DC/TAg group and on the other, significantly lower in comparison to the other DC-transduced groups (**Figure 1N**). In order to determine the ability of DCs with decreased expression of IL-10R to trigger a specific anti-tumor immune response, the co-culture of naïve lymphocytes and DCs was performed. Splenocytes activated by DC/shIL-10R_1/TAg eliminate cancer MC38 cells most efficiently, which was reflected in the highest cytotoxic activity in relation to other groups (**Figure 1O**). Based on the gathered data, in particular the relative expression of the *il10ra* gene and promotion of specific immune response, for further *in vivo* experiment, the shIL-10R_1 sequence was selected (named hereinafter as shIL-10R).

### 3.2 Therapy with immunomodulatory dose of HES-MTX nanoconjugate and DCs with downregulated IL-10R expression-based vaccines induced tumor growth inhibition in murine MC38 colon carcinoma model

**Figure 2:**
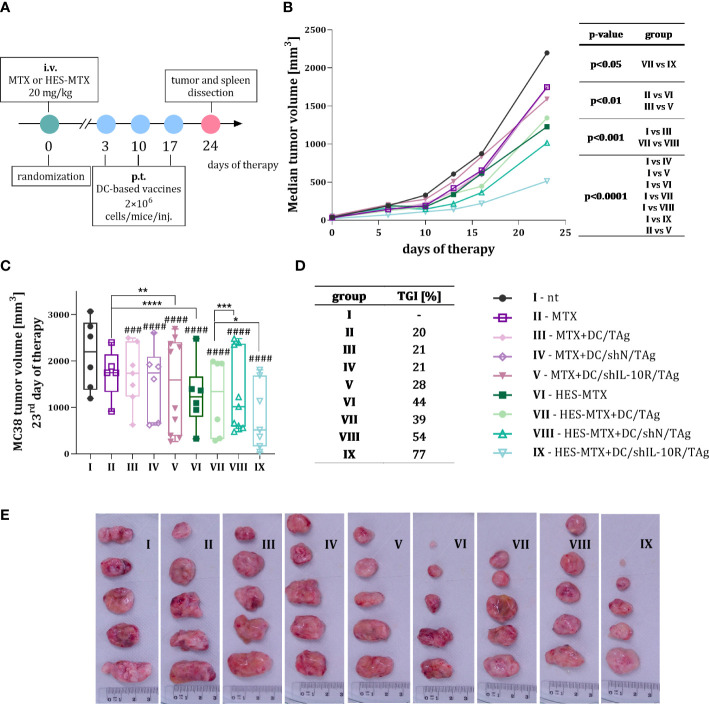
MC38 tumor growth after combined therapy with MTX or nanoconjugate HES-MTX followed by multiple injections of DC-based vaccines with downregulated IL-10R expression. **(A)** Scheme of treatment. **(B)** Graph presenting MC38 tumor growth kinetics after chemoimmunotherapy. **(C)** Box graph presenting tumor volume individual for every mouse in the group with the indicated median for each group on the 23^rd^ day of the experiment (5-10 mice per group). **(D)** Table with the percentage of MC38 tumor growth inhibition (TGI) calculated on the 23^rd^ day of the experiment in relation to the MC38 not treated (nt) group. **(E)** Images of the dissected MC38 tumor nodules from not treated and treated groups of mice (representative tumors obtained from five mice from each group). Results are expressed as median **(B)** and median with min to max values **(C)**. Differences between groups **(B, C)** were calculated using two−way ANOVA followed by Bonferroni’s multiple comparisons *post−hoc* test. The asterisks (*) presented in the graphs indicate statistically significant differences between the given groups; hashtags (#) above a bar indicate a statistically significant difference between the given group and the control group – nt (*p<0.05; **p<0.01; ***/###p<0.001; ****/####p<0.0001).

In the next step of the research, we decided to determine whether the modulation of immune response induced by the administration of MTX-based chemotherapeutic agents influences the effectiveness of DCs-based vaccines with downregulated IL-10R expression. For this purpose, mice with subcutaneously growing MC38 tumor intravenously received 20 mg/kg MTX or HES-MTX nanoconjugate, and three days later immunotherapy with DC-based vaccines was started (**Figure 2A**). Considering the therapeutic effect of applied therapy as a delay in tumor growth, it was shown that in comparison to MTX, the use of HES-MTX nanoconjugate contributes to substantial inhibition of MC38 growth, not only in the case of sole chemotherapy, but also when it was combined with immunotherapy (**Figures 2B–E**). It should be highlighted, that in MTX-treated group of mice we noted only minor inhibition of tumor development, regardless of whether MTX was used alone or in combination with DC-based vaccines. Moreover, the use of transduced DCs did not significantly improve the therapeutic activity of MTX.

It was observed that administration of transduced DCs after the application of HES-MTX, especially when DC/shIL-10R/TAg was used, caused the greatest slowdown of tumor growth – in HES-MTX + DC/shIL-10R/TAg group MC38 nodules were 77% smaller than in not treated (nt) control group (**Figure 2D**). In addition, to assess the impact of applied combined therapy on the changes in local and systemic anti-tumor immune response, seven days after the last injection of DC-based vaccines, the tumors and spleens were collected for further *ex vivo* analyses.

### 3.3 Combined therapy with HES-MTX and DC/shIL-10R/TAg resulted in favorable changes in the landscape of immune cells infiltrating MC38 tumors

**Figure 3:**
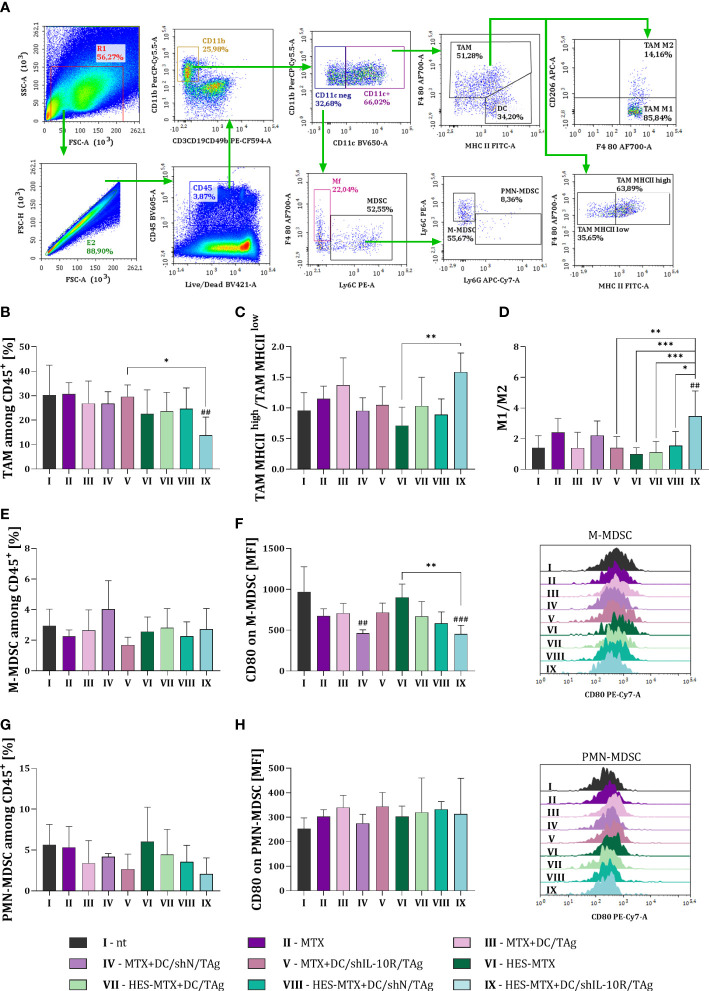
Impact of combined chemoimmunotherapy on MC38 tumor infiltration with myeloid immune cells. **(A)** Scheme of multiparameter flow cytometry analysis showing the method of distinguishing myeloid cell subpopulation in tumors dissected from MC38 tumor−bearing mice treated according to the scheme presented in **Figure 2A**. Green arrows indicate the subsequent stages of flow cytometric analysis. **(B)** Percentage of TAMs among CD45^+^ cells in tumors. **(C)** TAM MHC II^high^/TAM MHC II^low^ and **(D)** M1/M2 ratios showing the polarization of TAMs in MC38 tumor tissue. Percentage of **(E)** M-MDSCs and **(G)** PMN-MDSCs among CD45^+^ cells in tumor. Expression of CD80 on **(F)** M-MDSCs and **(H)** PMN-MDSCs surface presented as mean fluorescence intensity (MFI). The representative data for each group are shown in the overlay histograms. Results are expressed as mean + SD calculated for 5-8 mice per group. Differences between groups were calculated using **(C, D)** the Brown−Forsythe and Welch ANOVA test followed by Dunnett’s T3 multiple comparisons *post−hoc* test; **(B)** one−way ANOVA followed by Tukey’s multiple comparisons *post−hoc* test or **(F)** nonparametric Kruskal−Wallis test followed by Dunn’s multiple comparisons test. The asterisks (*) presented in the graphs indicate statistically significant differences between the given groups; hashtags (#) above a bar indicate a statistically significant difference between the given group and the control group - nt (*p<0.05; **/##p<0.01; ***/###p<0.001).

**Figure 4:**
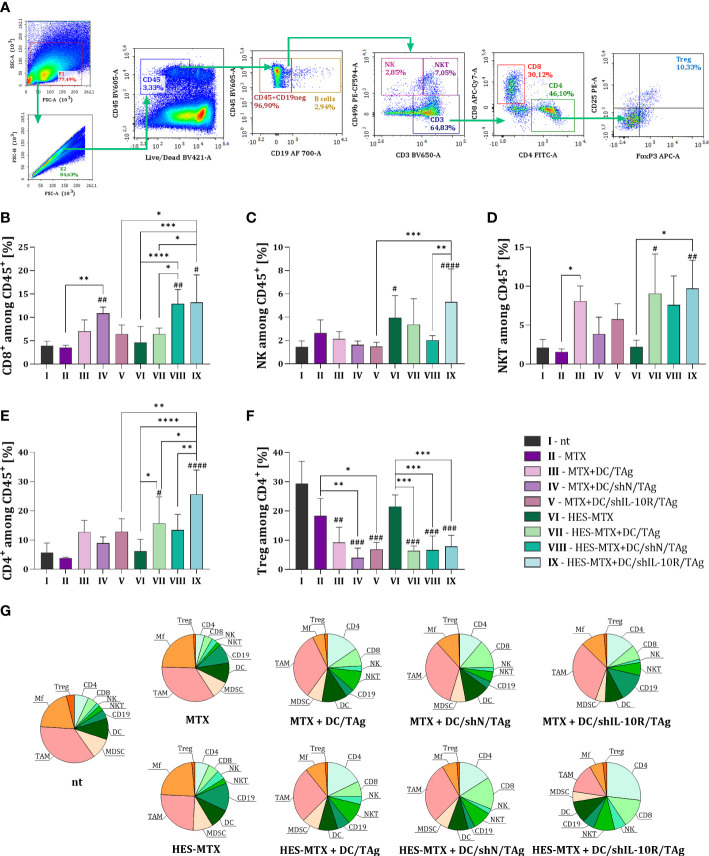
Influence of applied combined chemoimmunotherapy on the tumor-infiltration of lymphocytes and overall landscape of immune cells in MC38 tumors. **(A)** Scheme of multiparameter flow cytometry analysis showing the method of distinguishing lymphoid cell subpopulation in tumors dissected from MC38 tumor−bearing mice treated according to the scheme presented in **Figure 2A**. Green arrows indicate the subsequent stages of flow cytometric analysis. Percentage of **(B)** CD8^+^, **(C)** NK, **(D)** NKT and **(E)** CD4^+^ cells among CD45^+^ cells in tumors. **(F)** Percentage of T regulatory lymphocytes among CD4^+^ T cells in tumor tissue. Results are expressed as mean + SD calculated for 5-8 mice per group. **(G)** Pie charts presenting the each immune cell type as a percentage of CD45^+^ cells in MC38 tumors and changes in the size of these populations after applied therapy. Differences between groups were calculated using **(E, F)** the Brown−Forsythe and Welch ANOVA test followed by Dunnett’s T3 multiple comparisons *post−hoc* test; **(B, C)** one−way ANOVA followed by Tukey’s multiple comparisons *post−hoc* test or **(D)** nonparametric Kruskal−Wallis test followed by Dunn’s multiple comparisons test. The asterisks (*) presented in the graphs indicate statistically significant differences between the given groups; hashtags (#) above a bar indicate a statistically significant difference between the given group and the control group – nt (*/#p<0.05; **/##p<0.01; ***/###p<0.001; ****/####p<0.0001).

Flow cytometric analysis made it possible to identify multiple immune cell subpopulations among CD45^+^ leukocytes infiltrating tumor tissue (**Figures 3A**, **4A**). Although various populations of immune cells present in the tumor were assessed during the analysis, we decided to discuss selected results that indicate the impact of applied therapy on the changes in the landscape of TME. The percentage of tumor infiltrating macrophages – TAMs (CD45^+^CD11b^+^CD11c^+^F4/80^+^), as well as MHC II expression on their surface reflecting the activation level of this cell population was investigated. Additionally, based on CD206 intracellular expression in TAMs it was possible to calculate the M1/M2 ratio, which allowed to evaluate the direction of TAMs polarization. Another immune cell population determined in tumors were two distinct myeloid-derived suppressor cell (MDSCs) subpopulations – monocytic (M−)MDSCs (CD45^+^CD11b^+^CD11c^−^F4/80^−^Ly6C^+^Ly6G^−^) and polymorphonuclear (PMN−)MDSCs (CD45^+^CD11b^+^CD11c^−^F4/80^−^Ly6C^int^Ly6G^+^). Due to the suppressive activity of these cells, determination of the expression of the CD80 molecule on their surface was performed.

Combined therapy consisting of HES-MTX and DC/shIL-10R/TAg resulted in a significant decrease in the percentage of TAMs among CD45^+^ cells in tumor tissue (**Figure 3B**). Furthermore, considering the activation status of TAMs that remained in tumor nodules, it was observed that cells with high MHC II expression and M1-type TAMs were dominant among them (**Figures 3C, D**). The above changes indicate that the immunosuppressive role of TAMs was decreased only after the use of HES-MTX nanoconjugate and DCs with downregulated IL-10R expression, while in the case of the other therapeutic groups, including MTX-treated groups, such an effect did not occur. Although none of the applied therapy resulted in a reduced influx of M-MDSCs into tumors, the changes in the CD80 expression on their surface were reported. Nevertheless, the lowest expression of CD80 co-stimulatory molecule was observed in MTX + DC/shN/TAg and HES-MTX + DC/shIL-10R/TAg groups (**Figures 3E, F**). In all DC-treated groups of mice we observed not statistically reduction in the percentage of PMN-MDSCs, especially when DC/shIL-10R/TAg cells were used. However we did not notice any substantial changes in the CD80 expression on the PMN-MDSCs (**Figures 3G, H**).

The changes occurring in the myeloid immune cell populations were accompanied by modifications in the percentage of lymphoid cells infiltrating MC38 tumors. In the case of lymphoid cell subpopulation infiltrating into tumor tissue, the size of CD8 (CD45^+^CD3^+^CD8^+^), NK (CD45^+^CD49b^+^), NKT (CD45^+^CD3^+^CD49b^+^) and CD4 (CD45^+^CD3^+^CD4^+^) cells among leukocytes in the tumor were determined, according to the scheme presented on **Figure 4A**. Moreover, the percentage of regulatory T lymphocytes (CD45^+^CD3^+^CD4^+^CD25^+^FoxP3^+^) among CD4^+^ T cells was also investigated.

After the application of transduced DCs, especially in combination with HES-MTX, the highest influx of CD8^+^ T cells into the MC38 tumor was observed. It should be emphasized that, when comparing both DC/shIL-10R/TAg-treated groups of mice, the use of nanoconjugate in the therapy resulted in significant increase in the CD8^+^ T cell percentage (**Figure 4B**). A similar observation was made in the case of NK cells, when an increased percentage of those cells was noticed after administration of HES-MTX as sole chemotherapy or combined with DC/shIL-10R/TAg-based vaccines. Moreover, in this latter group this change was statistically significant when compared to MTX + DC/shIL-10R/TAg and HES-MTX + DC/shN/TAg groups (**Figure 4C**). The effect of DC-based vaccines on the increased tumor infiltration by effector immune cells was also reflected in the size of NKT cell and CD4^+^ T cell subpopulations, however, only in HES-MTX + DC/TAg and HES-MTX + DC/shIL-10R/TAg groups these alterations were statistically significant when compared to control (nt) group (**Figures 4D, E**). Similar to CD8^+^ T cells subpopulation, the treatment with nanoconjugate and DC/shIL-10R/TAg caused a significantly increased influx of CD4^+^ T cells into tumor tissue, not only in comparison to MTX + DC/shIL-10R/TAg group, but also in relation to other HES-MTX-treated groups of mice. It should be noted, that despite the fact that the administration of DC-based vaccines resulted in a substantial reduction in the percentage of Tregs among all CD4^+^ T cells present in tumors, the use of DCs with reduced sensitivity to IL-10 did not enhance this effect (**Figure 4F**). Thus, this observation suggests, that blockade of the negative impact of TME-derived IL-10 on DCs activity and function rather contributed to increase infiltration of effector lymphoid cells, than decrease the size of regulatory T cells subpopulation.

To summarize the results obtained after the flow cytometric analysis of MC38 tumors, we decided to present all analyzed tumor’ immune cells subpopulations as pie charts (**Figure 4G**). On their basis, it can be clearly seen that between groups of mice treated with MTX or HES-MTX, applied as sole chemotherapy or combined with DC-based vaccines, the essential changes in proportions of each analyzed immune cell subpopulations were observed only when nanoconjugate and DCs with downregulated IL-10R expression were used. In other types of combined therapy, although an increase in the size of the immunocompetent cell population (i.e. CD4, CD8, NK, NKT cells) was observed, there was an insufficient decrease in the population of immune cells with suppressor activity such as TAMs.

### 3.4 Changes induced by HES-MTX and DC/shIL-10R/TAg therapy at the local anti-tumor immune response level were crucial to enhance the effective specific immune response at the systemic level

**Figure 5:**
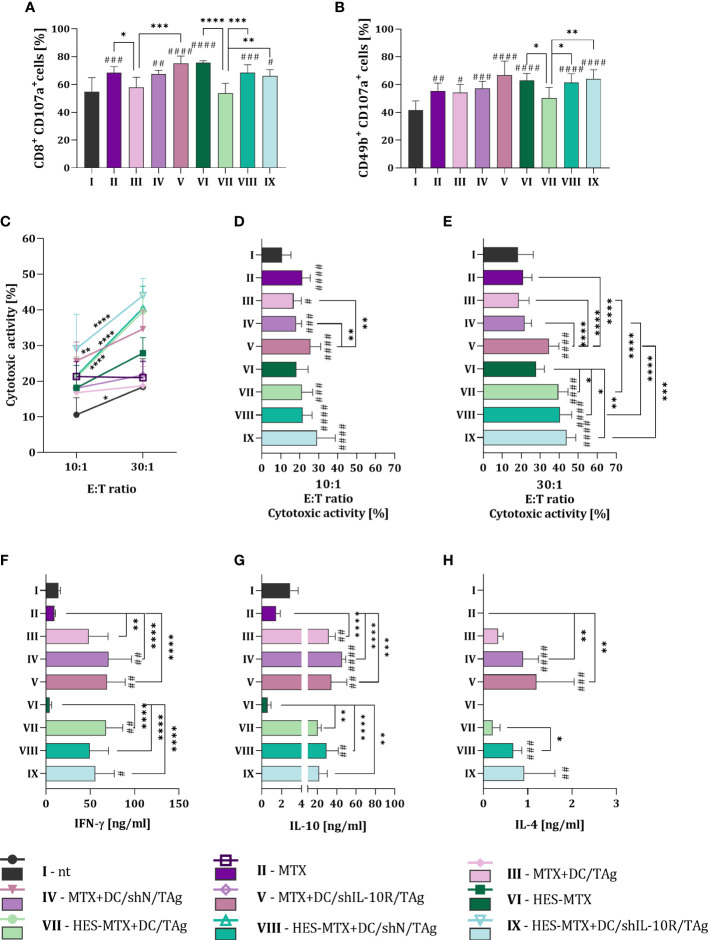
Effect of applied chemoimmunotherapy on induction of systemic anti-tumor response. Percentage of CD107a^+^ among restimulated **(A)** CD8^+^ and **(B)** NK (CD49b^+^) cells measured by degranulation assay. **(C-E)** Cytotoxic activity of restimulated splenocytes (effector cells) against DiO^+^ MC38 cells (target cells) after 4-hour incubation in 10:1 and 30:1 E:T ratios. **(F)** IFN-γ, **(G)** IL-10 and **(H)** IL-4 concentration in supernatants collected from the above splenocytes restimulated with MC38 cells. Results are expressed as mean + SD calculated for 5-8 mice per group. Differences between groups were calculated using **(D, E)** the Brown−Forsythe and Welch ANOVA test followed by Dunnett’s T3 multiple comparisons *post−hoc* test; **(A, B)** one−way ANOVA or **(C)** two−way ANOVA followed by Tukey’s multiple comparisons *post−hoc* test or **(F-H)** nonparametric Kruskal−Wallis test followed by Dunn’s multiple comparisons test. The asterisks (*) presented in the graphs indicate statistically significant differences between the given groups; asterisk above the line indicates statistical significance between different E:T ratios within a given group, while hashtags (#) above a bar indicate a statistically significant difference between the given group and the control group – nt (*/#p<0.05; **/##p<0.01; ***/###p<0.001; ****/####p<0.0001).

The final stage of research was the estimation of the impact of applied combined therapy on the systemic anti-tumor immune response. For this purpose, we conducted the five-days *ex vivo* restimulation of splenocytes obtained from MC38 bearing-mice receiving therapy. In this type of functional assay, we assessed the ability of splenocytes *in vivo* stimulated with tumor antigens, to activate a specific anti-tumor immune response after their secondary contact with MC38 cells. With the use of the degranulation assay, we determined the percentage of CD107a^+^ cells among the population of CD8^+^ and NK (CD49b^+^) cells. After treatment with MTX or HES-MTX used as a sole therapy or combined with vaccines based on transduced DCs the increase of CD8^+^CD107a^+^ cell percentage was observed. Although comparing the DC/TAg- with DC/shN/TAg- or DC/shIL-10R/TAg-treated groups of mice there were statistically significant changes in the population size of CD8^+^CD107a^+^ cells, it should be emphasized, that these alterations were not dependent on the of used chemotherapeutic, nor type of transduced DCs (**Figure 5A**). Similar observations were made in the population size of CD49b^+^CD107a^+^ cells – in almost all treated groups of mice (except HES-MTX + DC/TAg group), the increase in the percentage of CD107a^+^ among NK cells was found (**Figure 5B**). Nevertheless, considering the cytotoxic activity of restimulated splenocytes against MC38 cells, it can be noted that any type of combined therapy with DC/IL-10R/TAg resulted in the enhanced ability of effector cells to eliminate target cells. It should be highlighted that the greatest cytotoxic activity was observed in HES-MTX + DC/shIL-10R/TAg group (**Figures 5C–E**). Restimulated splenocytes obtained from immunotherapy-receiving mice were able to produce the cytokines in increased level when compared to control or chemotherapeutic groups. However, considering the decreased sensitivity of DCs to IL-10 present in the TME, we did not notice any effect of using the DC/shIL-10R/TAg-based vaccines on the changes in the IFN-γ and IL-10 production by splenocytes obtained from mice treated with this type of DCs. Only in the case of IL-4 concentration it can be assumed that after therapy with DC/shIL-10R/TAg, restimulated splenocytes were able to increase the secretion of IL-4, but these changes were not statistically significant (**Figures 5F–H**). However, it should be emphasized that even when restimulated spleen cells produced high amount of IL-4, IL-10 together with increased concentration of IFN-γ, the effect caused by HES-MTX + DC/shIL-10R/TAg therapy at the local anti-tumor immune response level, was strong enough to elicit the antigen-specific systemic immune response manifested by the greatest ability of splenocytes to eliminate cancer cells.

The all gathered data indicate that DCs with decreased sensitivity to the negative impact of TME-derived IL-10 are able to elicit the effective immune response, which led to the inhibition of tumor growth, but only when their administration is preceded by the use of chemotherapeutic with immunomodulatory properties. The beneficial changes in the MC38 tumors’ immune cells milieu were caused only when therapy with HES-MTX and DC/shIL-10R/TAg was applied. Furthermore, the highest cytotoxic activity of splenocytes obtained from mice treated with nanoconjugate and DC/shIL-10R/TAg was noted. This observation allowed us to postulate that the changes that have been triggered by such therapy at the local immune response level, have a decisive impact on the generation of efficient and specific anti-tumor response at the systemic level.

## 4 Discussion

In the present work we demonstrated for the first time that the application of cellular vaccines based on DCs with downregulated IL-10R expression preceded by the administration of HES-MTX nanoconjugate – the chemotherapeutic with immunomodulatory potential, resulted in the creation of an efficient and specific anti-tumor immune response leading to significant inhibition of tumor growth. Moreover, we were the first to apply chemotherapy with HES-MTX nanoconjugate together with cellular vaccines based on DCs with decreased IL-10R expression in the murine MC38 colon carcinoma model.

In our previous studies in the MC38 tumor model, we had shown the immunomodulatory potential of HES-MTX nanoconjugate (32). Three days after HES-MTX administration, the influx of CD8^+^ and NK cells into tumor tissue had increased and it had been accompanied by the elimination of cells with suppressor activity from TME. Furthermore, at the systemic anti-tumor immune response level, we had observed on the one hand – the enlargement of CD4^+^, CD8^+^ and NKT cell percentages among splenic leukocytes, and reduction in the size of Tregs population among CD4^+^ cells, on the other. Therapy with HES−MTX resulted in the increased cytotoxic activity of restimulated splenocytes. It should be added that none of these observations had been made in mice treated with MTX in the free, unconjugated form. Determining what changes had occurred after the use of nanoconjugate was very important because, in our treatment schedule in this tumor model, the immunotherapy with DC-based vaccines begins on the third day after chemotherapeutic administration. The knowledge about the direction of the anti-tumor response at this time point had allowed us to determine to which environmental conditions DCs will be applied, and to estimate the therapeutic effect of such combined therapy. Although after therapy consisting of HES-MTX and DC/TAg, an enhancement of the local and systemic anti-tumor immune response had been observed, however in the comparison to HES-MTX group of mice we had not observed the intensification of the therapeutic effect in the inhibition of the tumor growth, as we initially had expected (32).

Hence, we put forward a hypothesis that one of the factors which may have an adverse influence on the functions of DCs administered peritumorally as cellular vaccines, is IL-10 which is abundantly present in tumor tissue. This immunosuppressive cytokine can be produced not only by tumor cells themselves, but also by immune cells present in TME, such as TAMs, MDSCs and Tregs (6–8).

Taking into consideration our previous observations, in current research we focused on enhancing the therapeutic effect of combined therapy through the use of DCs with downregulated expression of IL-10 receptor – by such modification, cells should be able to initiate anti-tumor response in unfavorable environmental conditions. In addition, we paid attention to the influence of immunomodulation induced by different MTX-based chemotherapeutic agents on the efficiency of the immune response generated by these modified DCs.

Therefore, the first step of our research was aimed at selection the most effective shRNA sequence directed against the IL-10R gene. With the use of lentiviral vectors encoding the shRNA against the targeted sequence, the DCs with decreased IL-10R expression were obtained. Moreover, this change was accompanied by increased expression of costimulatory molecules on the surface of modified DCs, regardless of the used shIL-10R sequence. Similar observations were found in cells transduced with the control shRNA sequence (DC/shN/TAg) and should be explained by the immunogenicity of lentiviral vectors. Our observations are in line with the research of others indicating an elevated expression of these surface molecules on the DCs after lentiviral transduction (43, 44). Moreover, Breckpot et al. demonstrated that myeloid DCs recognize lentiviral antigens through TLR3 and TLR7 toll-like receptors, which leads not only to increased expression of MHC II and costimulatory molecules but also enhanced IFN-β production by these cells (44).

It should be highlighted that achieved by us the approx. 50% reduction of the relative level of IL-10R expression (in the DC/shIL-10R_1/TAg group) in relation to DC/TAg was sufficient to increase the ability of the modified DCs to activate a specific anti-tumor response. Naïve T-cells stimulated with DC/shIL-10R_1/TAg were characterized by the greatest cytotoxic activity, reflected in the highest percentage of dead MC38 cells – compared to DC/TAg and DC/shN/TAg cells. The above data are in agreement with the results obtained by Thepmalee et al., which found that T cells activated by DCs with half-decreased IL-10R expression showed increased cytolytic activity against cholangiocarcinoma cells compared to T cells stimulated by DCs transduced with control shRNA sequence (25). Similar observations were made when the anti-IL-10R antibody was used, which confirms that lowering the sensitivity of DCs to the influence of IL-10, significantly increases the effectiveness of created by DCs specific anti-tumor response (45). Moreover, Kim et al. proved that the therapy with DCs with downregulated IL-10R expression in the TC-1 tumor model resulted in strong CD8^+^ T cells antigen-specific response, which led to a significant slowdown in tumor growth (22). It has been shown by Qiao et al. that blocking the IL-10-signalling axis through the use of fusion protein targeting the EGFR and IL-10 (CmAb-(IL10)_2_) enabled to the abolition of the CD8^+^ T cells apoptosis by DCs in an IL-10R signal-dependent manner (46).

Therefore, based on data mentioned above published by others and results of our *in vitro* functional assays with the use of DC/shIL-10R_1/TAg, we postulated that this type of DC-based vaccines applied to MC38-tumor bearing mice will be able to trigger an efficient anti-tumor immune response, despite the presence of IL-10 in TME. Moreover, considering the aforementioned immunomodulatory potential of nanoconjugate, we assumed that the changes in the anti-tumor immune response that occurred after the use of HES-MTX will be crucial for the activation of an effective and specific immune response generated by DCs with decreased sensitivity to the negative impact of the IL-10.

Indeed, in the presented research, we observed the slowest growth of MC38 tumor in the group of mice treated with HES-MTX and DC/shIL-10R/TAg – median tumor volume was 77% smaller than in the control non-treated group. For comparison – in all MTX-treated groups, only minor inhibition of tumor development was noted, regardless of whether MTX was used alone or in combination with DC-based vaccines. These observations on the increased therapeutic efficacy of MTX conjugates are consistent with the research of others. For example, Ciekot et al. showed that, hydroxyethylcelullose-MTX conjugate significantly inhibited the growth of 4T1 murine breast tumor compared to MTX in free form (47). Similar observations were made by Woźniak et al., who found that the glucose-MTX conjugate was more effective than its unconjugated counterpart in delaying the development of 4T1 tumors (48). However, none of these referenced research specifies what changes in the anti-tumor response occurred after using the conjugates that could explain the observed results.

In our present studies, in the case of other HES-MTX-receiving groups of mice, we found that the use of nanoconjugate together with DC/TAg vaccines did not enhance the therapeutic efficacy in comparison to sole therapy with HES-MTX. It is consistent with our previous research (32) and it should be explained by suppressive factors present in the TME, such as IL-10, that adversely affect the DC/TAg-based vaccines. The confirmation of this assumption can be found not only in the calculated TGI value for therapy consisting of HES-MTX + DC/shIL-10R/TAg, but also in the changes in local and systemic anti-tumor response elicited by this type of treatment.

It should be highlighted that only in the HES-MTX + DC/shIL-10R/TAg group of mice the reduction in the size of the TAM population infiltrating tumor tissue was observed and this should be considered as a good prognostic marker (49). It is well known that suppressive properties of TAMs, such as IL-10 secretion and increased arginase 1 expression, are closely related to the inhibition of T cells activity and proliferation (50). Thus, the decrease in the TAMs percentage, accompanied by high expression of MHC II on remaining TAM [which are considered as more inflammatory type of cells (51, 52)], indirectly caused an increased accumulation of effector immune cells able to eliminate tumor cells. Moreover, only the abovementioned type of therapy (with nanoconjugate and DC with downregulated IL-10R expression) resulted in strong M1-type cells predomination, which indicates a favorable polarization of TAMs towards cells with strong anti-tumor properties, which stimulate a Th1-type response (53).

MDSCs are another immune cell population that significantly impacts on the quality of the anti-tumor response. These cells suppress the immune response, i.a., *via* interaction of their CD80 with Tregs’ CTLA-4 surface molecule (54). However, among two MDSC subpopulations present in tumors, M-MDSC cells have stronger immunosuppressive properties (55). It is also believed that M-MDSC cells are characterized by a greater ability to inhibit T cells activity than compared to the PMN-MDSCs. Accordingly, effective anti-tumor therapy should lead to the elimination of the M-MDSC subpopulation from TME (56). When assessing the size and activity of both MDSC subpopulations in MC38 tumors, a reduction in the percentage of M-MDSCs was not demonstrated, but the treatment consisting of HES-MTX + DC/shIL-10R/TAg resulted in a decrease in CD80 expression on their surface, indicating the reduced suppressor properties of these cells. On the other hand, in the case of the PMN-MDSCs subpopulation, a decrease in their percentage was found after the use of each type of DC-based vaccines, but their suppressor characteristics resulting from the expression of the CD80 molecule remained unchanged.

Hence, we postulate that the changes in myeloid immune cell populations which occurred after treatment with HES-MTX + DC/shIL-10R/TAg, were crucial to the creation of a favorable landscape of lymphoid immune cells infiltrating MC38 tumor tissue. More precisely – alterations in the size and activation status of TAMs and MDSCs contributed to an enhanced influx into tumor nodules of CD8^+^, NK, NKT and CD4^+^ cells. Moreover, an assessment of the effect of combined therapy on the cytotoxic activity of restimulated spleen cells found that, splenocytes collected from mice receiving HES-MTX + DC/shIL-10R/TAg eliminated MC38 tumor cells most efficiently. It is worth mentioning that these changes were significant in comparison to MTX + DC/shIL-10R/TAg. This indicates that the changes that have been induced at the local immune response level have a decisive impact on the formation of an effective specific anti-tumor response at the systemic level.

Therefore based on obtained results it can be summarized, that the fate of the immune response generated by DCs, may be closely related to the modulatory potential of the applied chemotherapeutic agent. The alterations in the landscape of immune cells that occurred after HES-MTX administration, which we confirmed in our previous research, were beneficial for the development of an efficient anti-tumor immune response by DCs applied as cellular vaccines, but only when these cells were properly prepared to action in adverse environmental conditions.

## 5 Conclusions

In conclusion, the downregulation of IL-10R expression in DCs enhances their ability to activate the specific anti-tumor immune response. However, the most significant factor that affects the DCs-generated immune response is immunomodulation induced by the chemotherapeutic. The use of HES-MTX nanoconjugate resulted in beneficial changes in TME and in the systemic immune response, which in turn enabled the development of an effective anti-tumor response initiated by DC/shIL-10R/TAg. All these factors contributed to a significant delay in the MC38 tumor growth.

## Data availability statement

The raw data supporting the conclusions of this article will be made available by the authors, without undue reservation.

## Ethics statement

The animal study was reviewed and approved by the Local Ethics Committee for Experiments with the Use of Laboratory Animals, Wrocław, Poland (authorization no. 31/2016 and 068/2020).

## Author contributions

Conceptualization, AS and EP-P; methodology, EP-P, JR, and TG; formal analysis, AS; investigation, AS, KW-C, AW, JM, JR, BS-O, NA-G, MŚ, and EP-P; data curation, AS; writing – original draft preparation, AS and EP-P; writing – review and editing, AS, KW-C, AW, JM, JR, BS-O, NA-G, MŚ, TG, and EP-P; visualization, AS; supervision, EP-P; project administration AS; funding acquisition, AS. All authors contributed to the article and approved the submitted version.
